# Preoperative hospital admission and complications following reverse total shoulder arthroplasty for proximal humerus fractures

**DOI:** 10.1016/j.jseint.2024.11.023

**Published:** 2025-01-02

**Authors:** Kenny Ling, Dmitriy Butsenko, James Gallagher, Rachel Loyst, Steven H. Liu, David E. Komatsu, Edward D. Wang

**Affiliations:** a Department of Orthopaedics, Stony Brook University, Stony Brook, NY, USA; b Renaissance School of Medicine at Stony Brook University, Stony Brook, NY, USA

**Keywords:** Reverse total shoulder arthroplasty, Urgent, Proximal humerus fracture, Complications

## Abstract

**Background:**

Total shoulder arthroplasty is an increasingly popular surgical treatment for degenerative diseases of the shoulder. The expansion of indications for reverse total shoulder arthroplasty (rTSA) to include proximal humerus (PHFs) fractures has led to rTSA being performed in the inpatient setting, which potentially limits the time for preoperative patient optimization and management. The purpose of this study was to investigate the 30-day postoperative complications associated with rTSA performed on patients requiring preoperative inpatient admission.

**Methods:**

The authors queried the American College of Surgeons National Surgical Quality Improvement Program database for all patients who underwent rTSA for PHF between 2015 and 2020. Patient demographics and comorbidities were compared between “admitted inpatient” and “from home” cohorts using bivariate logistic regression. Multivariate logistic regression, adjusted for all significantly associated patient demographics and comorbidities, was used to identify associations between admitted inpatient rTSA and postoperative complications.

**Results:**

Patient demographics and comorbidities that were significantly associated with admitted inpatient rTSA for PHF were age≥ 75 (*P* < .001), American Society of Anesthesiologists classification ≥3 (*P* < .007), congestive heart failure (*P* = .001), open wound/wound infection (*P* < .001), bleeding disorders (*P* < .001), and transfusion prior to surgery (*P* < .001). Multivariate analysis found admitted inpatient rTSA for PHF to be independently associated with blood transfusions (odds ratio 2.27, 95% confidence interval 1.66-3.09; *P* < .001) and nonhome discharge (odds ratio 2.70, 95% confidence interval 2.16-3.38; *P* < .001).

**Conclusion:**

Patients who underwent inpatient rTSA for PHF while admitted had higher rates of bleeding disorders and preoperative transfusion. Postoperatively, inpatient rTSA for PHF was independently associated with higher rates of blood transfusions and nonhome discharge within the 30-day postoperative period, compared to rTSA performed for PHF in patients presenting from home.

Total shoulder arthroplasty (TSA) is an increasingly popular surgical treatment for degenerative diseases of the shoulder. TSA includes both anatomic and reverse (rTSA) techniques. TSA has traditionally been indicated for the treatment of glenohumeral osteoarthritis.^8^ The development of rTSA has expanded indications to include failed TSA, rotator cuff arthropathy, primary glenohumeral osteoarthritis, and proximal humerus fractures (PHFs).^4^^,^^9^

Overall, TSA has been performed at an increasing rate over the past few decades.^2^^,^^6^^,^^10^^,^^12^ The incidence of TSA increased nearly 3-fold from 2005 to 2013.^6^ This rise is predicted to increase to more than 9-fold by 2030.^10^ This increase can largely be attributed to rTSA becoming more popular for the treatment of osteoarthritis, irreparable rotator cuffs and PHFs.^3^^,^^4^^,^^6^ A recent study found that rTSA for primary glenohumeral osteoarthritis with a preserved rotator cuff had similar outcomes to anatomic TSA and rTSA with cuff arthropathy.^9^ Studies have shown that rTSA has been increasingly performed over hemiarthroplasty for surgical treatment of PHFs.^13^

The purpose of this study was to investigate the 30-day postoperative complications associated with rTSA performed in the inpatient setting. We hypothesized that inpatient rTSA on admitted patients was associated with higher rates of surgical site infection and readmission.

## Methods

The authors queried the American College of Surgeons National Surgical Quality Improvement Program database for all patients who underwent TSA between 2015 and 2020. This study was rendered exempt from approval by our University’s Institutional Review Board, as the National Surgical Quality Improvement Program (NSQIP) database is fully deidentified. Data in the NSQIP database are obtained from over 600 hospitals in the United States and is collected by trained surgical clinical reviewers. The data are periodically audited to maintain high fidelity.

*Current Procedural Terminology* code 23472 was used to identify patients who underwent TSA, both anatomic and reverse, between 2015 and 2020. The exclusion criteria inherent to the NSQIP database automatically exclude all cases for patients younger than 18 years of age. The database also excludes cases classified as trauma that were performed emergently on the same day of admission. Cases were also excluded if any of the following variables had missing information: height/weight, discharge destination, American Society of Anesthesiologists (ASA) classification, functional health status, readmission status, and elective surgery status. Additionally, International Classification of Diseases 9 and 10 codes for postoperative diagnoses were used to exclude cases of TSA not performed for PHFs. As anatomic TSA is not commonly performed for PHF, the remaining cases were assumed to be rTSA.

Variables collected in this study included patient demographics, comorbidities, surgical characteristics, and 30-day postoperative complication data. Patient demographics included gender, body mass index, age, smoking status, functional status, ASA classification, and preoperative steroid use. Preoperative comorbidities included insulin-dependent and non–insulin-dependent diabetes, severe chronic obstructive pulmonary disease, congestive heart failure (CHF), hypertension, disseminated cancer, open wound/wound infection, bleeding disorders, and transfusion prior to surgery. Surgical characteristics included operative duration in minutes. Complications that occurred within 30 days postoperatively were included in the analysis. These complications included pneumonia, superficial incisional surgical site infection (SSI), deep incisional SSI, organ/space SSI, wound dehiscence, reintubation, pulmonary embolism, ventilator > 48 hours, urinary tract infection, stroke, cardiac arrest, myocardial infarction, blood transfusion, deep vein thrombosis, sepsis, septic shock, readmission, reoperation, nonhome discharge, and mortality.

The initial pool of patients was divided into 2 cohorts based on whether their procedure was classified as elective or urgent. By NSQIP definition, cases were considered elective if the patient was brought in from home on the day of the surgery and if the surgery was a nonemergent/nonurgent scheduled procedure. Patients were still considered to be presenting from “home” if they had to stay with friends or family, or a local hotel, due to logistical reasons. “Home” also included nursing home, assisted care facility, prison, or other nonhospital institution if that was the patient’s normal living environment. These cases were designated as the “from home” cohort. By NSQIP definition, cases were considered urgent if patients were inpatients at an acute care hospital, transferred from an emergency department, transferred from a clinic, underwent an emergent/urgent surgical case, or admitted to the hospital on any date prior to the procedure. These cases were designated as the “admitted inpatient” cohort.

A total of 27,050 patients underwent primary TSA in NSQIP from 2015 to 2020. Cases were excluded as follows: 152 for missing height/weight, 11 for missing discharge destination, 29 for missing ASA classification, 227 for missing functional health status prior to surgery, 2 for missing readmission status, and 5 for unknown elective surgery status. Additionally, 24,443 cases were excluded for postoperative diagnoses that were not PHFs. Of the 2181 patients remaining, 1630 (74.7%) underwent rTSA from home and 551 (25.3%) underwent rTSA while admitted inpatient.

All statistical analyses were conducted using SPSS Software (version 29.0; IBM Corp., Armonk, NY, USA). Patient demographics and comorbidities were compared between cohorts using bivariate logistic regression. Multivariate logistic regression, adjusted for all significantly associated patient demographics and comorbidities, was used to identify associations between urgent rTSA and postoperative complications. Odds ratios (ORs) were reported with 95% confidence intervals (CIs). The level of statistical significance was set at *P* < .05.

## Results

Patient demographics and comorbidities that were significantly associated with admitted inpatient rTSA for PHF were age ≥ 75 (*P* < .001), ASA classification ≥ 3 (*P* = .007), CHF (*P* = .001), open wound/wound infection (*P* < .001), bleeding disorders (*P* < .001), and transfusion prior to surgery (*P* < .001 (Table I).

**Table I:** Patient characteristics for patients who underwent rTSA while admitted inpatient vs. scheduled electively.

Characteristic	From home		Admitted inpatient		*P* value
	Number	Percent	Number	Percent	
Total	1630	100.0%	551	100.0%	
Age					**<.001**
18-39	6	0.4%	0	0.0%	
40-64	645	39.6%	187	33.9%	
65-74	346	21.2%	91	16.5%	
≥75	633	38.8%	273	49.5%	
Body mass index (kg/m^2^)					.524
<18.5	35	2.1%	9	1.6%	
18.5-29.9	839	51.5%	268	48.6%	
30.0-34.9	380	23.3%	147	26.7%	
35.0-39.9	199	12.2%	76	13.8%	
≥40.0	177	10.9%	51	9.3%	
Gender					.205
Female	1327	81.4%	435	78.9%	
Male	303	18.6%	116	21.1%	
Functional status					.066
Independent	1546	94.8%	511	92.7%	
Dependent	84	5.2%	40	7.3%	
ASA classification					**.007**
1-2	567	34.8%	157	28.5%	
≥3	1063	65.2%	394	71.5%	
Diabetes mellitus					.427
No	1236	75.8%	407	73.9%	
Noninsulin	235	14.4%	87	15.8%	
Insulin	159	9.8%	57	10.3%	
Current smoker					.110
No	1403	86.1%	489	88.7%	
Yes	227	13.9%	62	11.3%	
COPD					.496
No	1500	92.0%	512	92.9%	
Yes	130	8.0%	39	7.1%	
Congestive heart failure					**.001**
No	1613	99.0%	534	96.9%	
Yes	17	1.0%	17	3.1%	
Hypertension					.138
No	544	33.4%	165	29.9%	
Yes	1086	66.6%	386	70.1%	
Disseminated cancer					.347
No	1614	99.0%	548	99.5%	
Yes	16	1.0%	3	0.5%	
Open wound/wound infection					**<.001**
No	1618	99.3%	536	97.3%	
Yes	12	0.7%	15	2.7%	
Chronic steroid use					.722
No	1553	95.3%	527	95.6%	
Yes	77	4.7%	24	4.4%	
Bleeding disorders					**<.001**
No	1573	96.5%	504	91.5%	
Yes	57	3.5%	47	8.5%	
Transfusion prior to surgery					**<.001**
No	1620	99.4%	525	95.3%	
Yes	10	0.6%	26	4.7%	
Operative duration (min)					.236
≤79	202	12.4%	72	13.1%	
80-128	754	46.3%	238	43.2%	
≥129	674	41.3%	241	43.7%	

*ASA*, American Society of Anesthesiologists; *COPD*, chronic obstructive pulmonary disease; *rTSA*, reverse total shoulder arthroplasty.

Bold *P*-values indicate statistical significance with *P* < .05.

Bivariate analysis was used to identify postoperative complications associated with admitted inpatient rTSA for PHF (Table II). The 30-day postoperative complications that were significantly associated with inpatient TSA were pneumonia (*P* = .050), urinary tract infection (*P* = .012), blood transfusions (*P* < .001), and nonhome discharge (*P* < .001).

**Table II:** Bivariate analysis of 30-day postoperative complications in patients who underwent rTSA while admitted inpatient vs. scheduled electively.

Complication	From home		Admitted inpatient		*P* value
	Number	Percent	Number	Percent	
Pneumonia	15	0.92%	11	2.00%	**.050**
Superficial incisional SSI	3	0.18%	0	0.00%	.999
Deep incisional SSI	3	0.18%	1	0.18%	.990
Organ/Space SSI	8	0.49%	1	0.18%	.347
Wound dehiscence	2	0.12%	2	0.36%	.278
Reintubation	9	0.55%	5	0.91%	.502
Pulmonary embolism	7	0.43%	2	0.36%	.834
Ventilator >48 hrs	5	0.31%	3	0.54%	.431
Urinary tract infection	15	0.92%	13	2.36%	**.012**
Stroke	2	0.12%	1	0.18%	.749
Cardiac arrest	2	0.12%	1	0.18%	.383
Myocardial infarction	10	0.61%	7	1.27%	.138
Blood transfusions	112	6.87%	95	17.24%	**<.001**
Deep vein thrombosis	11	0.67%	2	0.36%	.418
Sepsis	5	0.31%	4	0.73%	.198
Septic shock	1	0.06%	0	0.00%	1.000
Readmission	80	4.91%	34	6.17%	.251
Reoperation	54	3.31%	19	3.45%	.879
Nonhome discharge	312	19.14%	238	43.19%	**<.001**
Mortality	7	0.43%	3	0.54%	.730

*rTSA*, reverse total shoulder arthroplasty; *SSI*, surgical site infection.

Bold *P* values indicate statistical significance with *P* < .05.

After adjusting for all significantly associated patient variables, multivariate logistic regression identified the 30-day postoperative complications associated with admitted inpatient rTSA for PHF (Table III). Multivariate analysis found admitted inpatient rTSA to be independently associated with blood transfusions (OR 2.27, 95% CI 1.66-3.09; *P* < .001) and nonhome discharge (OR 2.70, 95% CI 2.16-3.38; *P* < .001). The remaining complications were no longer significant after adjustment.

**Table III:** Multivariate analysis of 30-day postoperative complications in patients who underwent rTSA while admitted inpatient vs. scheduled electively.

Complication	Odds ratio	95% CI	*P* value
Pneumonia	1.60	0.69-3.67	.265
Urinary tract infection	2.14	0.99-4.64	.053
Myocardial infarction	1.93	0.72-5.15	.188
Blood transfusions	2.27	1.66-3.09	**<.001**
Nonhome discharge	2.70	2.16-3.38	**<.001**

*rTSA*, reverse total shoulder arthroplasty; *CI*, confidence interval.

Bold *P* values indicate statistical significance with *P* < .05.

## Discussion

In this study, we reported 30-day postoperative complications associated with rTSA for PHF on admitted inpatients using a large national database. Based on NSQIP’s definition, urgent (admitted inpatient cohort) cases were defined as patients in the inpatient setting or transferred from the emergency department. Elective (from home cohort) cases were defined as patients presenting to surgery from home or an outpatient setting. Our analysis included 2181 patients with PHF, of which 1630 (74.7%) underwent rTSA from home and 551 (25.3%) underwent rTSA while admitted inpatient. Through multivariate analysis, we identified inpatient TSA for PHF to be independently associated with higher rates of postoperative blood transfusions and nonhome discharge. This was not surprising as the admitted inpatient cohort was found to be associated with higher rates of bleeding disorders and preoperative transfusion.

In our study, we found that the admitted inpatient rTSA cohort had a greater proportion of patients of age >75 and higher ASA classification. Furthermore, the admitted inpatient rTSA cohort was associated with higher rates of CHF, open wound infection, bleeding disorders, and transfusion prior to surgery. This intuitively makes sense, as patients with open wounds or patients requiring transfusion would necessitate hospital admission for stabilization and surgery for fracture management while inpatient. Open wounds were defined in NSQIP as a breach of the integrity of the skin > 2 cm, and therefore open wounds do not necessarily equate to open fracture. Studies by Crespo et al and Paras et al found that rTSA procedures performed for proximal humerus fractures were strongly associated with older female patients.^5^^,^^11^ Moreover, older postmenopausal women are associated with osteoporosis and a greater risk of fracture, particularly those with reduced physical function.^1^ While a majority of the patients were females over the age of 75, there was no significant difference in age or gender between the home cohort and admitted inpatient cohort.

A similar study by Crespo et al used a prospectively collected, single-institution database to compare rTSA for PHFs vs. elective rTSA for nonfracture indications in 1984 patients. This study found that rTSA for PHFs was associated with a longer hospital length of stay and greater intraoperative blood loss. However, the authors reported no significant difference in adverse intraoperative events.^5^ While our study compared only cases with fractures, our findings that inpatient rTSA is associated with higher rates of nonhome discharge and blood transfusion align with their observations of greater length of stay and intraoperative blood loss.

Overall, we found admitted inpatient rTSA yielded more 30-day postoperative complications compared to from home rTSA. Given that rTSA has shown to provide favorable long-term outcomes in the treatment of PHFs, our findings are not meant to discourage the use of rTSA in the acute inpatient setting.^7^^,^^14^^,^^15^ Rather, our study highlights the postoperative complications that are more prominent in the acute inpatient setting to better equip surgeons in optimizing perioperative care. It is also important to recognize that although “urgent” surgery was defined in NSQIP by location from where patients presented rather than by time to surgery. Therefore, differences in surgeon and institutional practices/preferences for admitting patients prior to surgery may have affected the cohorts. Further studies may look at the time from initial injury to surgery as a risk factor for complications.

This study was limited to the information available from the NSQIP database. Therefore, we could not account for operative variables such as the institution where the procedure was performed, experience of the surgeon, and postoperative rehabilitation. Relating specifically to our findings, postoperative transfusion may be dependent on bleeding disorders, physician tolerances, anticoagulant use, and axillary artery injury. We were unable to account for preoperative or intraoperative axillary artery injury, which may increase the need for postoperative transfusion. Furthermore, we were limited to postoperative complications that occurred within 30 days of the surgery and were unable to consider long-term postoperative complications such as fracture, instability, and loosening. Finally, the coding for the initial data input for the NSQIP database may be a significant limitation, as many of the postoperative diagnoses were unrelated to orthopedic issues. Therefore, patients with PHFs may have been missed if PHF was not the primary postoperative diagnosis for the patient.

## Conclusion

Admitted inpatient rTSA performed for the treatment of PHF was independently associated with higher rates of postoperative blood transfusions and nonhome discharge within the 30-day postoperative period, compared to electively scheduled rTSA. The indications for rTSA have expanded and now include an older population with more chronic comorbidities. While preoperative assessment may be limited in cases performed in the acute inpatient setting, this study may better equip surgeons for optimizing care postoperatively.

## Disclaimers:

Funding: No funding was disclosed by the authors.

Conflicts of interest: The authors, their immediate families, and any research foundation with which they are affiliated have not received any financial payments or other benefits from any commercial entity related to the subject of this article.
